# APX3330 Promotes Neurorestorative Effects after Stroke in Type One Diabetic Rats

**DOI:** 10.14336/AD.2017.1130

**Published:** 2018-06-01

**Authors:** Tao Yan, Poornima Venkat, Michael Chopp, Alex Zacharek, Peng Yu, Ruizhuo Ning, Xiaoxi Qiao, Mark R. Kelley, Jieli Chen

**Affiliations:** ^1^Gerontology Institute, Neurology, Tianjin Medical University General Hospital, Tianjin Neurological Institute, Key Laboratory of Post-Neurotrauma Neurorepair and Regeneration in Central Nervous System, Ministry of Education and Tianjin City, Tianjin, China; ^2^Department of Neurology, Henry Ford hospital, Detroit, MI, USA; ^3^Department of Physics, Oakland University, Rochester, MI, USA; ^4^Department of Neurology, First Hospital Harbin, Harbin, China.; ^5^Department of Ophthalmology, Henry Ford Hospital, Detroit, MI, USA; ^6^Herman B Wells Center for Pediatric Research, Indiana University School of Medicine, Indianapolis, IN, USA

**Keywords:** Stroke, Type 1 Diabetes Mellitus, APX3330, neuroprotection, neurorestoration

## Abstract

APX3330 is a selective inhibitor of APE1/Ref-1 redox activity. In this study, we investigate the therapeutic effects and underlying mechanisms of APX3330 treatment in type one diabetes mellitus (T1DM) stroke rats. Adult male Wistar rats were induced with T1DM and subjected to transient middle cerebral artery occlusion (MCAo) and treated with either PBS or APX3330 (10mg/kg, oral gavage) starting at 24h after MCAo, and daily for 14 days. Rats were sacrificed at 14 days after MCAo and, blood brain barrier (BBB) permeability, ischemic lesion volume, immunohistochemistry, cell death assay, Western blot, real time PCR, and angiogenic ELISA array were performed. Compared to PBS treatment, APX3330 treatment of stroke in T1DM rats significantly improves neurological functional outcome, decreases lesion volume, and improves BBB integrity as well as decreases total vessel density and VEGF expression, while significantly increases arterial density in the ischemic border zone (IBZ). APX3330 significantly increases myelin density, oligodendrocyte number, oligodendrocyte progenitor cell number, synaptic protein expression, and induces M2 macrophage polarization in the IBZ of T1DM stroke rats. Compared to PBS treatment, APX3330 treatment significantly decreases plasminogen activator inhibitor type-1 (PAI-1), monocyte chemotactic protein-1 and matrix metalloproteinase 9 (MMP9) and receptor for advanced glycation endproducts expression in the ischemic brain of T1DM stroke rats. APX3330 treatment significantly decreases cell death and MMP9 and PAI-1 gene expression in cultured primary cortical neurons subjected to high glucose and oxygen glucose deprivation, compared to untreated control cells. APX3330 treatment increases M2 macrophage polarization and decreases inflammatory factor expression in the ischemic brain as well as promotes neuroprotective and neurorestorative effects after stroke in T1DM rats.

Hyperglycemia and insulin resistance are major risk factors for ischemic stroke, atherosclerosis, and vascular and cardiac complications [1]. Diabetes mellitus (DM) instigates a cascade of events which lead to vascular endothelial cell dysfunction, increased vascular permeability, a disequilibrium of angiogenesis, and poor recovery after ischemic stroke [2, 3]. Both stroke and diabetes cause extensive damage to white matter (WM) [4, 5] which can potentially lead to long term disability due to the brain’s limited capacity of axonal regeneration, and an unfavorable environment for axon regrowth, sprouting and remyelination in the ischemic brain [6]. Therefore, in addition to neuroprotection in the gray matter, therapies that protect WM and promote WM remodeling after stroke, will enable long term neurological functional recovery via strengthening residual neuronal connections, forging new connections, and axonal rewiring in the injured brain [7]. Approximately 30% of all stroke patients suffering from diabetes, battle worse prognosis at least in part due to high inflammation and exacerbated WM injury in the ischemic diabetic brain. Hence, there is a critical need to develop therapeutic strategies specifically to improve neurological functional outcome after stroke in the DM population.

In diabetic stroke, exacerbated blood brain barrier (BBB) disruption and inflammation are key factors that contribute to poor outcome [4, 8, 9]. Matrix metalloproteinases (MMP) are associated with BBB disruption, and known to aggravate WM injury [4, 8, 9]. Myelin degradation following ischemic stroke is attenuated in MMP9 knockout mice [10], implicating MMP’s in post-stroke WM injury. Inflammatory factors such as the receptor for advanced glycation endproducts (RAGE) play a vital role in promoting inflammation and diabetic complications [11, 12]. In our previous study, we have reported that RAGE is increased in diabetic stroke animals [13]. Increased RAGE expression has been implicated in promoting inflammation in the ischemic brain, and has been associated with neurological deficits in DM animals after stroke [13]. A permeable BBB facilitates the infiltration of peripheral macrophages into the ischemic brain [14, 15], and these macrophages initially assume an anti-inflammatory M2 phenotype and aid in clearing cellular debris from the injured brain [16, 17]. These macrophages can also assume/transition into a neurodegenerative M1 phenotype and hinder recovery by releasing proinflammatory factors. These pro-inflammatory molecules can in turn worsen BBB damage, initiate the recruitment of peripheral immune cells into the cerebral microcirculation and ischemic brain, as well as induce systemic inflammation [18]. Therefore, in diabetic subjects, it is important to control post stroke inflammatory responses and extend the M2 phase of macrophages and delay their transition into the pro-inflammatory M1 phenotype in to improve stroke outcome.

APX3330 is a small molecule inhibitor of apurinic apyrimidinic endonuclease redox effector factor-1 (APE1/Ref-1) redox activity [19]. Alterations in the expression, subcellular localization and activity of APE/Ref-1 have been associated with several diseases such as neurodegeneration, cancer and cardiovascular diseases. The role of APE1/Ref-1 in disease pathology and its potential as a novel therapeutic target has been reviewed elsewhere [19-21]. Several studies have demonstrated the specificity of APX3330 for APE1 [19]. APE1 activates nuclear factor kappa B (NFκB) and hypoxia-inducible factor 1α (HIF-1α), which are involved in apoptosis, inflammation and angiogenesis [20, 22]. NFκB is induced in neurons following stroke and is a key mediator of inflammatory responses and infarction development/expansion after stroke [23]. NFκB is also known to critically regulate resident inflammatory brain cells and microglia that are activated in response to ischemic injury [24]. In T1DM stroke rats, suppressing NFκB signaling pathway significantly decreases inflammatory factors such as RAGE and TLR4 expression in the ischemic brain and improves stroke outcome [25]. HIF-1α is a transcriptional activator that maintains oxygen homeostasis and functions as a master regulator of tissue responses to hypoxic conditions. While neuroprotective therapies that up regulate HIF-1α activity have been reported to promote pro-angiogenic responses and improve stroke outcome in diabetic mice [26], several reports also indicate that DM up regulates HIF-1α protein levels and increases HIF-1α transcriptional activity in endothelial cells, which in turn can adversely affect tight junction proteins and BBB permeability [27, 28]. DM drives increased but dysfunctional neovascularization in the cerebrovasculature, which upon ischemic stress can become leaky and disrupt/aggravate BBB rupture [29]. Hypoxia stimulates angiogenesis mainly via activation of HIF-1α and vascular endothelial growth factor (VEGF). In T1DM stroke rats, an abnormal and persistent increase in VEGF correlated with increased BBB permeability in the ischemic brain [30], and inhibiting VEGF improved tight junction proteins and decreased endothelial leakage i.e. BBB leakage [28].

APX3330 inhibits NFκB and HIF-1α activity [31], and can potentially attenuate neuroinflammatory responses and post ischemic angiogenesis, which makes APX3330 a suitable candidate for stroke therapy in DM rats. In this study, we are the first to investigate whether APX330 treatment could improve functional outcome after stroke in Type one diabetic (T1DM) rats, whether APX3330 induces neurorestorative effects, and what mechanisms are involved in APX330 treatment induced beneficial effects in T1DM stroke rats.

## MATERIALS AND METHODS

All experiments followed the standards of the American Council on Animal Care and Institutional Animal Care and Use Committee of Henry Ford Health System.

### T1DM induction

Adult male Wistar rats (225-250g, Jackson Laboratory) were induced with T1DM using a single intraperitoneal injection of Streptozotocin (STZ, 60 mg/kg) [32]. After 2 weeks, rats with fasting blood glucose >300 mg/dl were defined as T1DM and included in the study.

### Middle cerebral artery occlusion (MCAo) model and experimental groups

T1DM rats were anesthetized with 2% isoflurane in a chamber for pre-anesthetic, and spontaneously respired with 1.5% isoflurane in 2:1 N_2_O:O_2_ mixture using a facemask connected and regulated with a modified FLUOTEC 3 Vaporizer (Fraser Harlake). Rectal temperature was maintained at 37°C throughout the surgical procedure using a feedback regulated water heating system. T1DM rats were subject to transient (2 hour) MCAo via intraluminal vascular occlusion as previously described [33, 34]. T1DM stroke rats were randomized (n=7/group) and treated with phosphate buffered saline (PBS-vehicle control) or APX3330 (10 mg/kg, gavage) initiated at 24 hours after MCAo daily for 14 days. Rats were sacrificed at 14 days after MCAo for immunostaining quantification analysis.

### Functional tests

An investigator blinded to the experimental groups performed a battery of functional tests including the modified neurological severity score and foot-fault tests prior to MCAo, and on days 1, 7 and 14 after MCAo, as previously described [35]. The modified neurological severity score is a composite of motor, sensory, reflex and balance tests, with scores ranging between 0 (no deficit) to 18 (maximum deficit). Hence, a higher score indicates greater neurological severity. Rats with scores <6 (possibly small to no lesion, their condition improves regardless of treatment) and >13 (very large lesion, poor survival, their condition deteriorates regardless of treatment) were excluded before treatment. The foot-fault test is routinely employed to evaluate sensorimotor function, motor coordination and limb placement deficits during locomotion [36]. The rats were placed on a 2.5 cm × 2.5 cm grid floor (45 cm × 30 cm) placed 2.5 cm above the base floor. A foot-fault was defined as a fall or slip through a grid opening due to an inaccurate forelimb placement. Data are presented as the percentage of foot-faults of the left paw over a 100-forelimb movement.

### Immunohistochemical assessment

All animals were transcardially perfused with 0.9% saline, and brains were rapidly removed, and immersion fixed in 4% paraformaldehyde and then embedded in paraffin. Seven coronal brain sections were processed and a series of 6 μm thick sections were cut from blocks which were obtained from the center of the lesion (bregma -1 mm to +1 mm). Every 10^th^ coronal section for a total of 5 sections was used for immunohistochemical staining. Hematoxylin and Eosin (H&E) staining was used for ischemic lesion volume calculation and presented as a percentage of the lesion compared with the contralateral hemisphere [37]. Antibody against α-smooth muscle actin (αSMA, smooth muscle cell marker, mouse monoclonal IgG 1:800, Dako), Von Willebrand Factor (vWF, an endothelial cell marker, 1:400; Dako), VEGF (1:50, Santa Cruz), synaptophysin (synaptic protein marker, 1:500, Chemicon), neuron-glial antigen 2 (NG2, oligodendrocyte progenitor cell marker, 1:800, Chemicon) and 2′,3′-cyclic-nucleotide 3′-phosphodiesterase (CNPase, oligodendrocyte marker, 1:200, Chemicon), ED1 (microglia/macrophages marker, 1:30; AbD Serotec), and CD163 (M2 macrophage marker, 1:500, Abcam) were employed. FITC-Albumin (1:500, Abcam) staining was employed to evaluate BBB permeability, and Luxol fast blue (LFB) staining was used to demonstrate myelin. In control experiments, primary antibody was replaced by non-immune serum.

### Quantification analysis

An investigator blinded to the experimental groups performed all immunostaining quantification analysis. Five slides from each brain (each slide containing 8 fields from striatum of the ischemic boundary zone (IBZ)) were digitized under a 20× objective (Olympus BX40) using a 3-CCD color video camera (Sony DXC-970MD) interfaced with an MCID image analysis system (Imaging Research). The number of arteries stained with αSMA were counted and analyzed with regard to small and large vessels (≥10μm diameter). For other immunostaining measurements, positive cell numbers were counted for each field of view or the positive stained areas were measured using the densitometry function (MCID image analysis system) with a density threshold above unstained set uniformly for all groups [38-40].

### Angiogenic ELISA array

An additional set of rats (n=4/group) were sacrificed at 14 days after MCAo and brain tissue was extracted from the ischemic brain for angiogenic ELISA assays and Western blot assays. An angiogenesis array kit (R&D Systems, mouse angiogensis array kit) was used to simultaneously assess the relative levels of angiogenesis-related proteins in ischemic brain tissue which had been treated with or without APX3330. Briefly, samples were mixed with detection antibodies for 1 hour at room temperature and added to the array membrane. Then, the membranes were incubated over night at 2-8°C on a shaker. Following a washing step, horseradish peroxidase-conjugated streptavidin was added and incubated for 30 minutes. After washing, X-ray film and ImageJ analysis software were used to quantify array signals.

### Western blot assay

Equal amounts of cell lysate were subjected to Western blot analysis which was performed as previously described [41]. Specific proteins were visualized using a Super Signal West Pico chemiluminescence kit (Pierce). Protein concentration was measured using the BCA kit (Thermo Scientific). The following primary antibodies were used: anti-β-actin (1:2000; Santa Cruz Biotechnology), anti-MMP9 (1:500; Santa Cruz Biotechnology) and anti-RAGE (1:500, R&D Biosystems).

### Primary cortical neuron (PCN) culture

PCNs were harvested from pregnant (day 18) embryonic Wistar rats (Charles River). The cultures were prepared as previously described with some modifications [42, 43]. Briefly, the embryo cerebral cortex was dissected and dissociated in Ca2+ and Mg2+ free HBSS with 0.125% trypsin. The cells were placed on poly-d-lysine (Sigma) coated dishes (35 mm, Corning) and initially cultured in DMEM media (Life Technologies) with 5% fetal bovine serum for 5 hours, then neurobasal growth medium (Life Technologies) with 2% B-27 (Life Technologies), 2 mM GlutaMax, and 1% antibiotic-antimycotic was used.

**Figure 1.**
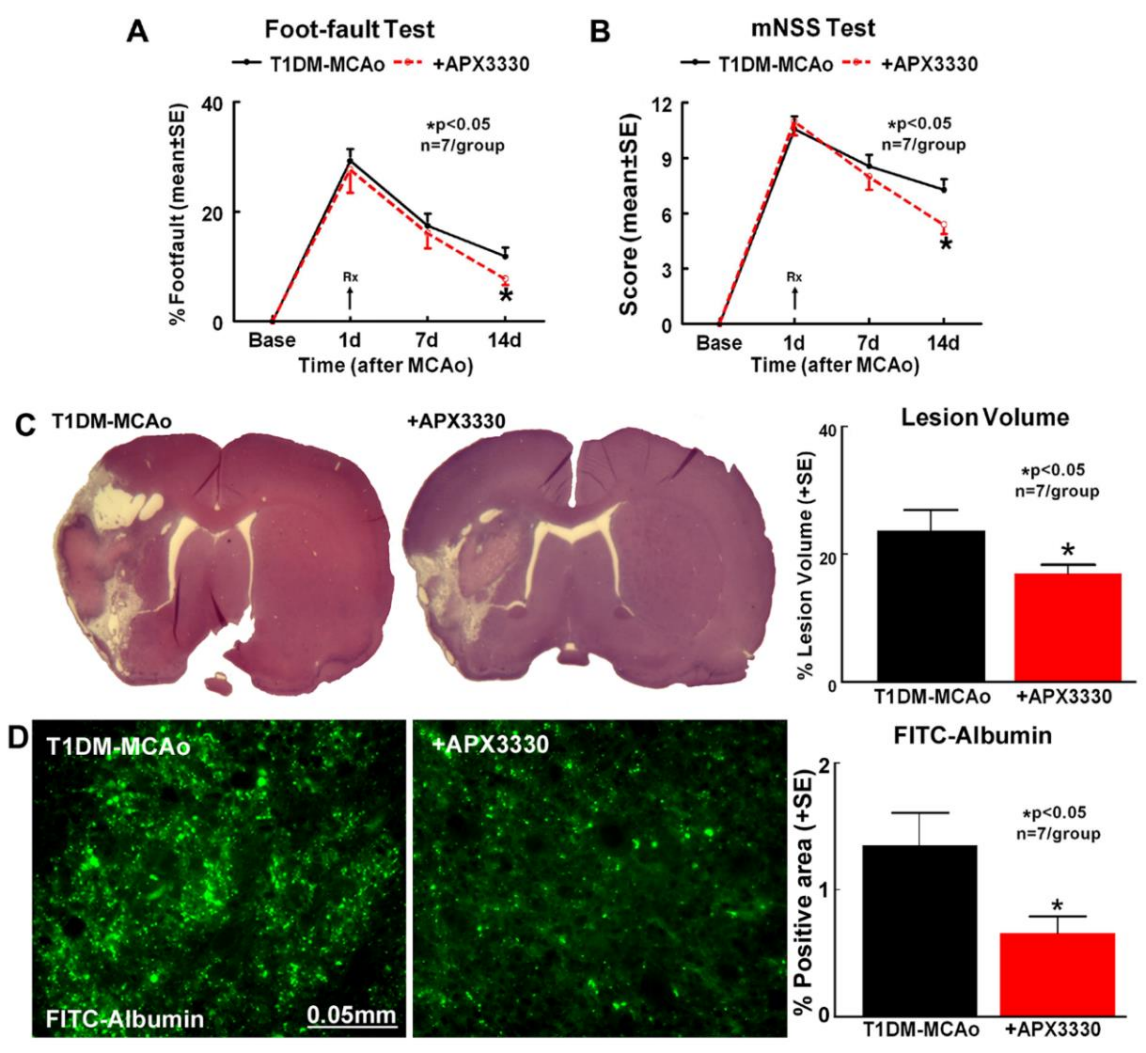
APX3330 improves stroke outcome and decreases ischemic burden and BBB permeability in T1DM rats. Treatment with APX3330 significantly improves neurological functional outcome after stroke in T1DM rats as indicated by (A) Foot-fault test and (B) modified neurological severity score (mNSS). APX3330 treatment also significantly decreases (C) ischemic lesion volume and (D) BBB disruption after stroke in T1DM rats.

### High glucose and oxygen glucose deprivation (OGD) conditions

To subject cells cultures to OGD, serum and glucose free media was used. Cells were placed in a hypoxia chamber (Forma Anaerobic System; Thermo Scientific) with 37°C incubator for 2 hours. After 2 hours, the cells were removed and replaced in high glucose cultured media (37.5 µM).

### Lactate dehydrogenase (LDH) assay

The CytoTox 96 non-radioactive cytotoxicity assay kit (Promega) was used following standard protocol. Briefly, cells were cultured in a 96 well plate for 24 hours and then the cells in half the wells for each experimental group were lysed to give total LDH, while media only was collected from the other set of wells for secreted LDH. The media was added to a fresh 96 well plate, followed by the addition of substrate solution. Following incubation, absorbance was recorded at 490 nm.

### MTS assay

3-(4,5-dimethylthiazol-2-yl)-5-(3-carboxy-methoxy-phenyl)-2-(4-sulfophenyl)-2H-tetrazolium (MTS) assay was performed using CellTiter 96 Aqueous One Solution Cell Proliferation Assay (Promega). 5000 cells were plated and 20 μL MTS was added to each well and incubated after treatment. Absorbance was recorded at 490 nm.

### Statistical analysis

All measurements and analyses were performed by normality of distribution. One-way Analysis of Variance (ANOVA) was used for the evaluation of functional tests, immunostaining, respectively. “Contrast/estimate” statement was used to test the group difference. All data are presented as mean ± standard error (SE).

**Figure 2.**
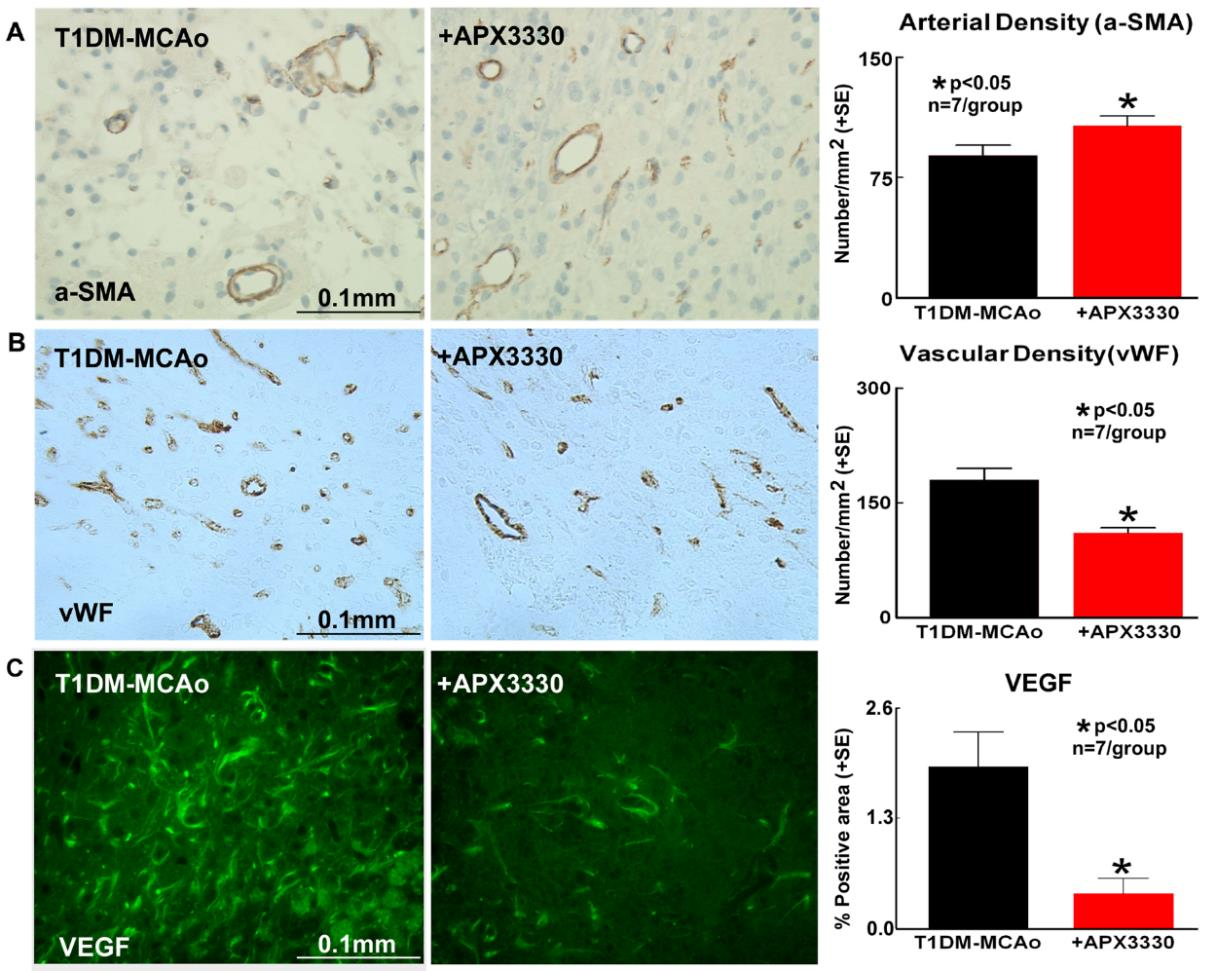
APX3330 increases arterial density and decreases dysfunctional angiogenesis after stroke in T1DM rats. APX3330 significantly increases (A) arterial density and decreases (B) vascular density and (C) VEGF expression in the ischemic border zone after stroke in T1DM rats, as indicated by α-smooth muscle actin (α-SMA), Von Willebrand Factor (vWF) and vascular endothelial growth factor (VEGF) immunostaining and quantification data respectively.

**Figure 3.**
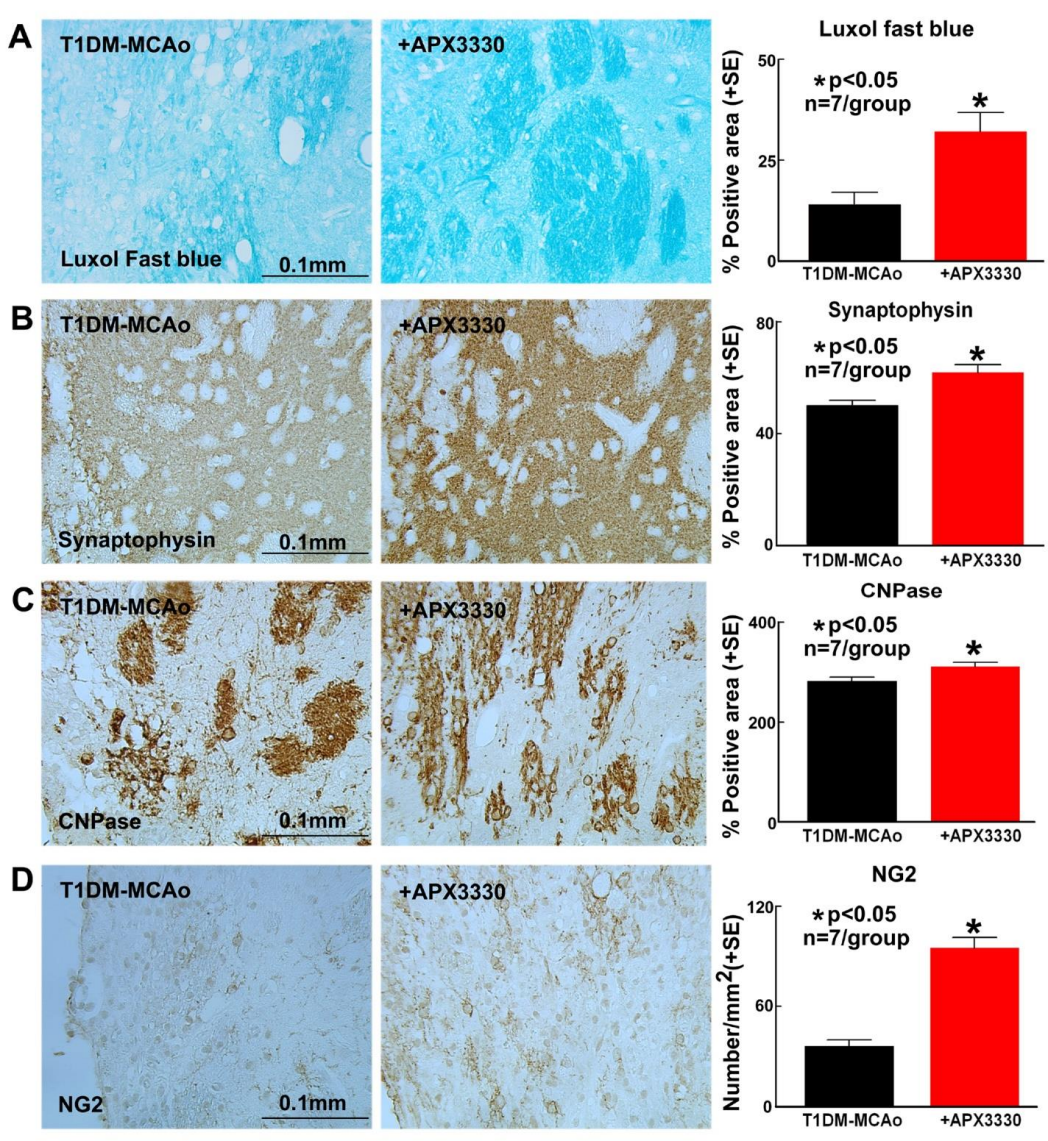
APX3330 significantly promotes white matter remodeling after stroke in T1DM rats. APX3330 significantly increases (A) myelin density (Luxol fast blue), (B) synaptic protein expression (Synaptophysin), (C) oligodendrocyte cell number (CNPase), and (D) oligodendrocyte progenitor cell (NG2) number in the ischemic border zone of T1DM stroke rats.

## RESULTS

### APX3330 treatment significantly improves neurological outcome, and decreases lesion volume and BBB permeability after stroke in T1DM rats

To evaluate the therapeutic effects of APX3330 treatment in T1DM stroke rats, mNSS and foot-fault tests were used to evaluate neurological function. APX3330 treatment significantly improves functional outcome after stroke in T1DM rats and the data show that the overall group effect was significant on day 14 after stroke (p<0.05, n=7/group, Fig. 1A-B). APX3330 significantly decreases ischemic lesion volume (p<0.05, n=7/group, Fig. 1C) and decreases BBB leakage (p<0.05, n=7/group, Fig. 1D) compared to PBS treated control T1DM stroke rats.

### APX3330 treatment decreases dysfunctional angiogenesis and VEGF expression in T1DM stroke rats

To test whether APX3330 treatment regulates post stroke angiogenesis, vascular and arterial density was measured in the IBZ. Figure 2A shows that APX3330 significantly increases arterial density (p<0.05, n=7/group) compared to PBS treated control T1DM stroke rats. However, total vascular density was significantly reduced in APX3330 treatment rats compared with PBS treated control T1DM stroke rats (p<0.05, n=7/group, Fig. 2B). APX3330 also significantly decreases VEGF expression in the IBZ (p<0.05, n=7/group, Fig. 2C) compared to PBS treated control T1DM stroke rats.

### APX3330 treatment significantly promotes WM and axonal remodeling after stroke in T1DM rats

To test whether APX3330 treatment regulates WM and axonal remodeling, Luxol fast blue (myelin density) and Synaptophysin (synaptic protein expression) expression were quantified in the IBZ. APX3330 significantly promotes myelin density (p<0.05, n=7/group, Fig. 3A) and synaptophysin expression (p<0.05, n=7/group, Fig. 3B) in the IBZ compared to PBS treated control T1DM stroke rats. APX3330 treatment also significantly increases oligodendrocyte (CNPase, p<0.05, n=7/group, Fig. 3C) and oligodendrocyte progenitor cell density (NG2, p<0.05, n=7/group, Fig. 3D) in the IBZ compared to PBS treated control T1DM stroke rats.

**Figure 4.**
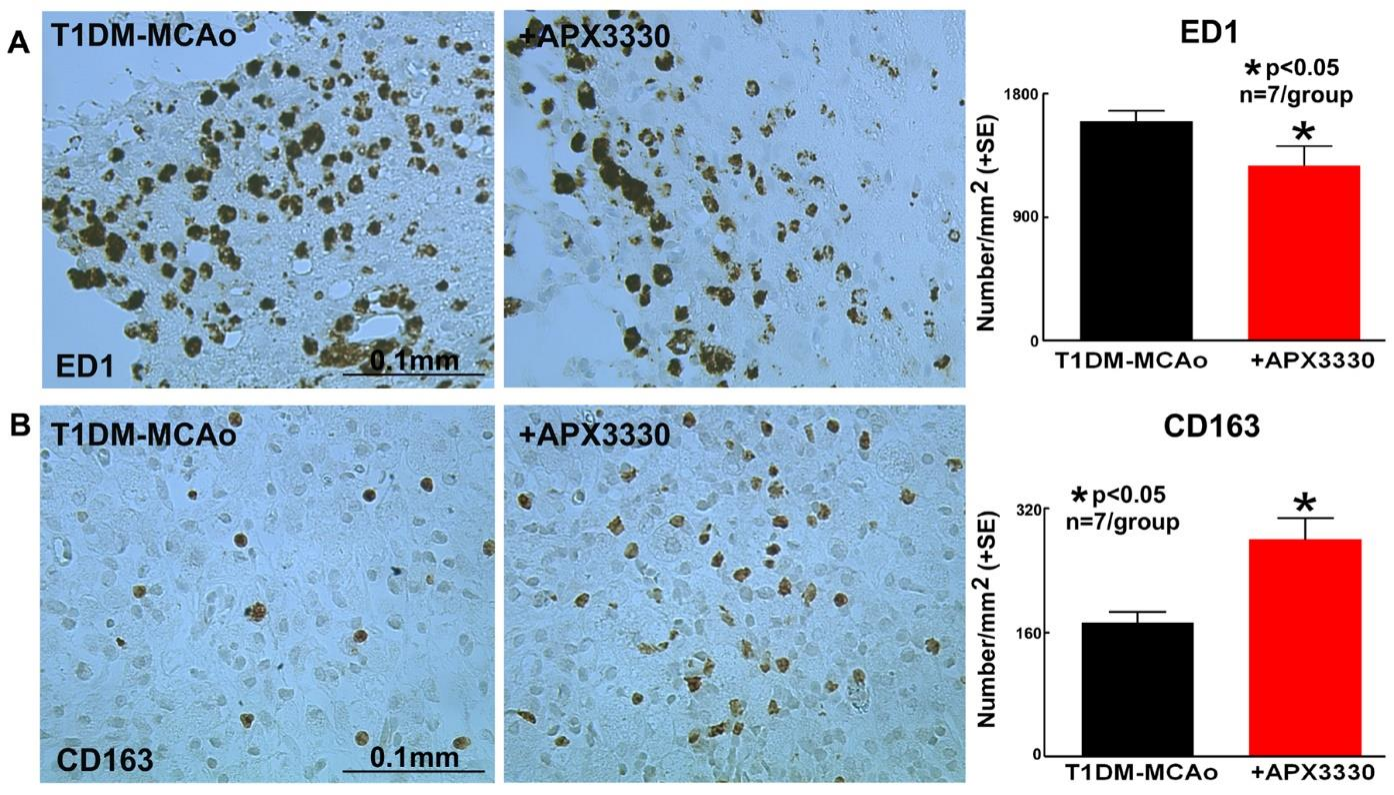
APX3330 treatment significantly promotes M2 macrophage polarization in the ischemic brain of T1DM stroke rats. APX3330 significantly decreases (A) ED1 (M1 macrophage marker, and significantly increases (B) CD163 (M2 macrophage marker) in the ischemic border zone of T1DM stroke rats.

### APX3330 treatment significantly decreases inflammatory factor expression after stroke in T1DM rats

**Figure 5.**
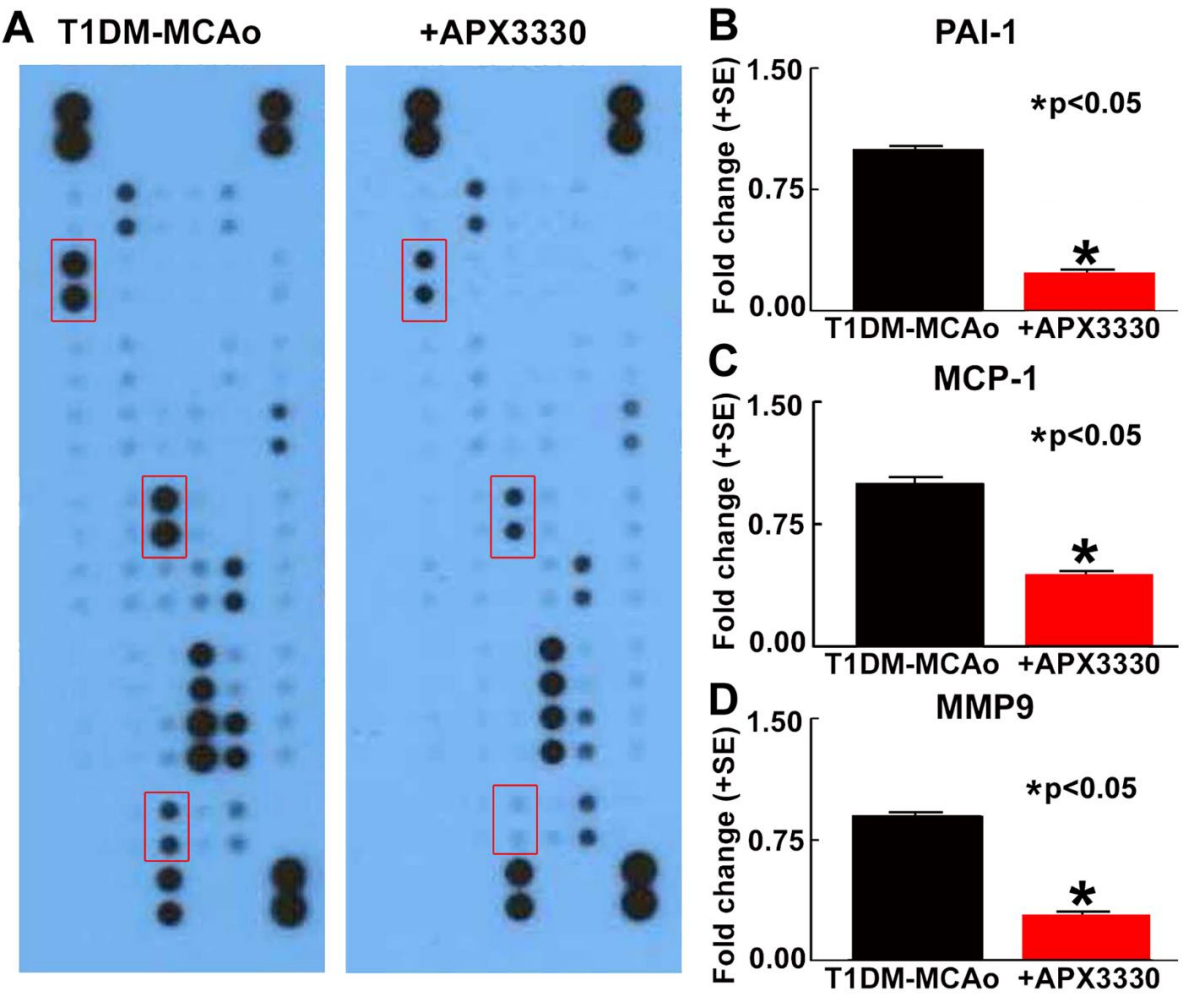
APX3330 treatment significantly decreases inflammatory factors after stroke in T1DM rats. (A) An ELISA array was employed to measure inflammatory protein expression in the ischemic brain. APX3330 significantly decreases (B) plasminogen activator inhibitor type 1 (PAI-1), (C) monocyte chemotactic protein-1 (MCP1) and (D) MMP9 compared to control T1DM stroke rats.

**Figure 6.**
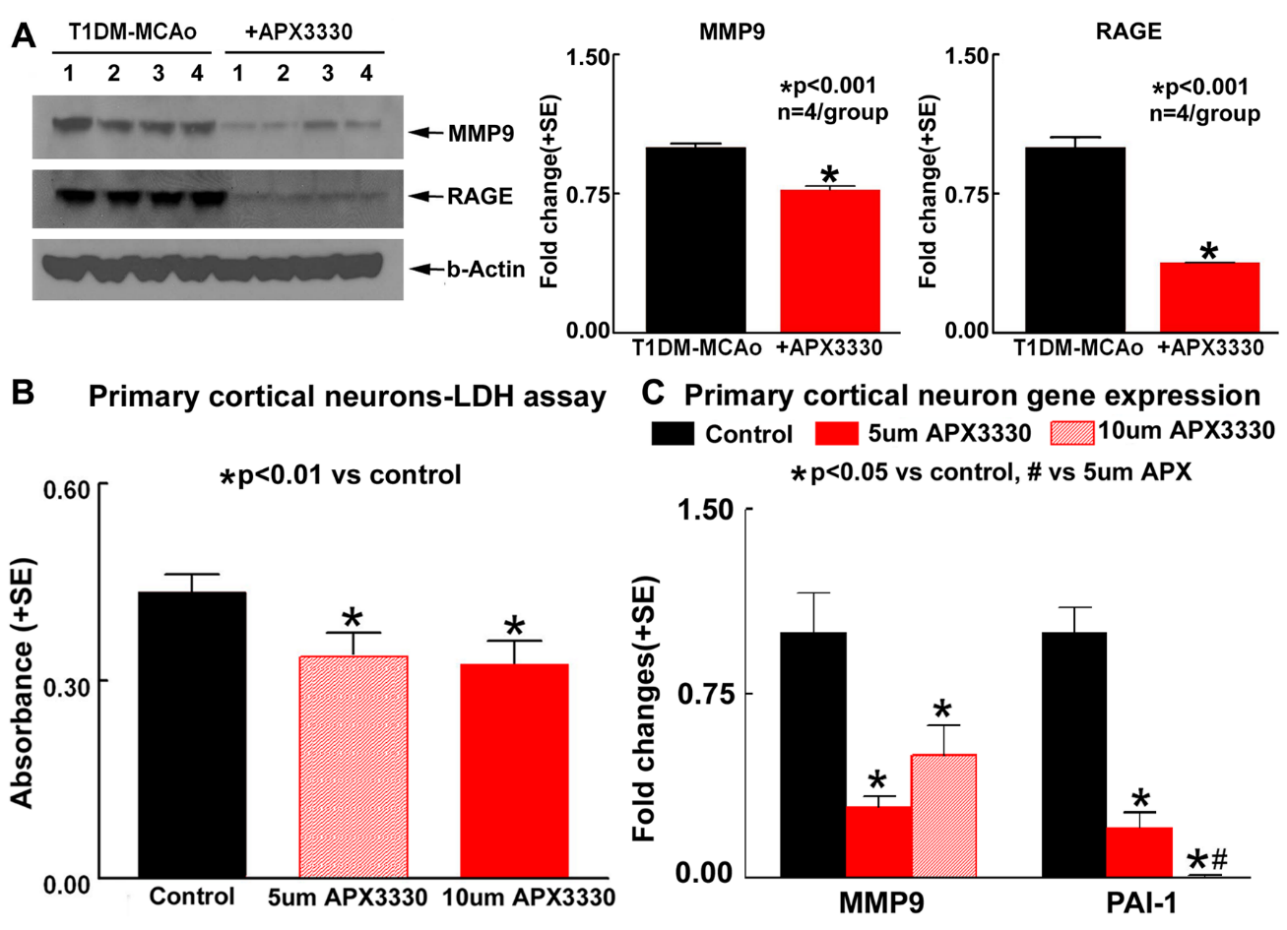
APX3330 treatment decreases inflammatory factor expression in the ischemic brain of T1DM stroke rats and significantly decreases primary cortical neurons cell death and inflammatory factor expression after stroke. (A) Western blot assay shows that APX3330 significantly decreases ischemic brain tissue expression of MMP9 and RAGE compared to control T1DM stroke rats. APX3330 treatment significantly decreases (B) primary cortical neuron cell death compared to control (untreated) cells as measured by LDH assay and (C) significantly decreases MMP9 and PAI-1 gene expression in primary cortical neurons subjected to high glucose and oxygen glucose deprivation, when compared to control (untreated) cells.

To investigate mechanisms of APX3330 induced neuroprotective and neurorestorative effects in T1DM stroke, we evaluated inflammatory factor expression in the ischemic brain. APX3330 treatment significantly promotes M2 macrophage polarization as indicated by decreased ED1 (M1 macrophage marker) and increased CD163 (M2 macrophage marker) in the IBZ of T1DM stroke rats (p<0.05, n=7/group, Fig. 4A-B). Angiogenic ELISA assay (Fig. 5A) shows that APX3330 decreases plasminogen activator inhibitor type 1 (PAI-1, 5-fold, Fig. 5B), monocyte chemotactic protein-1 (MCP1, 3-fold, Fig. 5C) and MMP9 (3-fold, Fig. 5D) compared to T1DM-MCAo control rats. We also measured the ischemic brain expression level of MMP9 and RAGE by Western blot. APX3330 significantly decreases ischemic brain tissue expression of MMP9 and RAGE compared to PBS treated control T1DM stroke rats (p<0.001, n=4/group, Fig. 6A). These data indicate that APX3330 treatment decreases inflammatory factor and pro-thrombotic factor expression in the ischemic brain after stroke in T1DM rats.

### APX3330 significantly decreases cell death and inflammatory factor gene expression in primary cortical neurons subjected to high glucose and OGD conditions

To test the effects of APX3330 on primary cortical neurons cell death and proliferation, LDH and MTS assays were performed. APX3330 treatment does not increase primary cortical neuron proliferation, but significantly decreases cell death compared to control (untreated) cells as measured by MTS (data not shown) and LDH (Fig. 6B) assays, respectively, which is consistent with the myelin protective effect observed in vivo. APX3330 treatment significantly decreases MMP9 and PAI-1 gene expression in primary cortical neurons subjected to high glucose and oxygen glucose deprivation, when compared to control (untreated) cells (Fig. 6C).

## DISCUSSION

In this study, we are the first to demonstrate that APX3330 treatment significantly improves neurological functional outcome and decreases lesion volume and BBB permeability after stroke in T1DM rats. APX3330 promotes WM remodeling and exerts myelin protective effects in the IBZ of T1DM stroke rats. By employing an angiogenic ELISA assay and Western blot analysis, we also confirmed that APX3330 treatment significantly decreases the expression of inflammatory factors such as MCP1, MMP9 and RAGE and pro-thrombotic factor PAI-1, in the ischemic brain of T1DM stroke rats. APX3330 significantly decreases gene expression of inflammatory factor MMP9 and pro-thrombotic PAI-1 in cultured primary cortical neurons subjected to high glucose and oxygen glucose deprivation conditions. These data indicate that APX3330 may be a novel treatment for T1DM stroke.

DM stroke induces extensive vascular damage, and our previous study has shown that there is a significant increase in vascular density in the ischemic border zone after stroke in T1DM rats compared to wild type non-DM rats [44]. However, the increase in vascular density is often an increase in non-functional blood vessels that contribute to brain hemorrhage and worse functional outcome after stroke [44]. Our present study shows that APX3330 treatment significantly increases arterial density but decreases total vascular density, indicating the potential of APX3330 to reverse DM stroke induced growth of unnecessary and non-functional blood vessels. BBB function is widely attributed to proper function and integrity of endothelial cells, tight junction protein and pericytes [45]. Aggravated BBB disruption has been demonstrated in T1DM stroke rats compared to non-DM stroke rats [44]. Protecting/improving BBB integrity is important for recovery after stroke, and we found that APX3330 decreases BBB leakage compared to control T1DM stroke rats.

In addition to massive vascular destruction, DM stroke also induces significant damage to the WM in the ischemic brain. Extensive WM after stroke has been associated with greater mortality, worse neuronal dysfunction and greater residual sensory-motor deficits after DM stroke [5, 46-48]. Promoting WM remodeling essentially initiates rewiring of the neural circuits thereby improving intercellular connectivity and communication. Since APX3330 significantly improves neurological function after stroke in T1DM rats, we investigated APX3330 effect on WM remodeling. Our data show that APX3330 significantly increases myelin density in the ischemic border zone of T1DM stroke rats compared to control T1DM stroke rats. In the central nervous system, oligodendrocyte and oligodendrocyte progenitor cells form myelin and exert crucial roles in WM function and homeostasis [49]. In several neurological diseases including stroke, WM damaged has been associated with oligovascular uncoupling [49]. Therefore, maintenance/increase of oligodendrocytes and oligodendrocyte progenitor cells number may contribute to WM recovery and decrease neurological functional deficits [49-51]. We found that APX3330 treatment in T1DM stroke rats significantly increases the number of oligodendrocytes and oligodendrocyte progenitor cells in the ischemic border zone, which may contribute to functional recovery after stroke in T1DM rats. Taken together, it is most likely that APX3330 improves neurological functional outcome via promoting WM remodeling after stroke in T1DM rats.

Neuroinflammatory responses play an important role in post-stroke recovery. While mild to moderate inflammation can be beneficial to brain repair, uncontrolled inflammation can create an inhospitable environment for brain repair and hinder/worsen recovery [7, 52]. In our study, Western blot analysis and an angiogenic ELISA array show that APX3330 treatment significantly decreases inflammatory factors such as MCP1, MMP9 and RAGE compared to control T1DM stroke rats. APX3330 also significantly decreases MMP9 and PAI-1 gene expression in cultured primary cortical neurons subjected to high glucose and OGD conditions. Both in-vivo after ischemic stroke and in-vitro using OGD model of stroke with primary rat cortical neurons, it has been reported that MMP9 is activated and expressed in neuronal nuclei and associated with delayed neuronal death [53]. Other studies have also reported that MMP9 inhibition in cultured primary cortical neurons subject to OGD decreases apoptosis and induces neuroprotective effects [54]. DM is associated with diminished fibrinolytic capacity, increased coagulability and increased PAI-1 concentration in blood [55]. Increased PAI-1 levels in the DM population can cause formation of intravascular thrombi and atherosclerotic lesions which in turn can cause stroke [56, 57]. Increased PAI-1 levels can trigger several cytokines and chemokines, and promote pro-inflammatory responses [58]. In addition, proinflammatory cytokines can disturb vascular endothelial function and increase PAI-1 level [59]. Elevated PAI-1 levels has also been suggested to be a true indicator of metabolic syndrome [59]. DM patients also have elevated MCP levels [60], which has been implicated in DM induced vascular calcification and diabetic nephropathy [61, 62]. Rats with nonfunctional MCP1 have smaller infarct volume and lesser macrophage infiltration post stroke, which suggests that attenuation of MCP1 may exert neuroprotective effects [63].

Inflammatory factors such as MMP9 and RAGE are typically increased in diabetic stroke animals [13]. In addition to regulating post stroke inflammatory responses, MMP9 also plays an important role in post stroke angiogenesis and BBB disruption [5, 64]. Increased MMP9 expression in the acute phase of stroke in DM patients creates a pro-inflammatory state that worsens WM damage. Neuroinflammation plays a major role in WM damage and oligodendrocyte death, in particular increased MMP9 expression has been associated with myelin breakdown [65, 66]. RAGE, as a proinflammatory factor, plays a critical role in neurodegenerative pathology, necrotic cell death and inflammatory responses [67]. The RAGE signaling pathway is involved in numerous pathological process following ischemia, such as inflammation, vascular injury, and brain damage [68, 69]. Our previous studies have implicated increased RAGE expression in worsening the outcome of DM stroke [13]. In this study, we found that APX3330 significantly decreases PAI-1, MCP-1, MMP9 and RAGE levels in the ischemic brain which may contribute to APX3330 induced neuroprotective and neurorestorative effects in T1DM stroke rats.

Following ischemic injury, macrophages promote both injury and repair depending on their phenotype. M2 macrophage polarization is a potential target for stroke therapy since anti inflammatory M2 macrophages exert neuroprotection, improve neuronal survival and decrease the expression of inflammatory factors in the ischemic brain [17, 70]. M2 macrophage polarization has been associated with improved functional recovery after stroke [71], as well as decreased neuroinflammation and enhanced axon growth in injured mouse spinal cord [72]. APX3330 promotes M2 macrophage polarization, marked by increased M2 macrophage marker CD163 and decreased M1 macrophage marker, ED1 expression in the IBZ of T1DM stroke rats. MCP-1 is a chemokine that regulates the migration and infiltration of macrophages in pathological tissue [73], and MCP-1-deficient mice subjected to stroke exhibit a reduction of phagocytic macrophage accumulation in the infarcted brain tissue [74]. Macrophage differentiation into proinflammatory M1 phenotype is mediated at least in part by RAGE/NF-κB pathway activation [75]. M1 Macrophages (in addition to monocytes and leukocytes) are a prolific source of inflammatory factors like MMP9 [76]. Therefore, it is likely that APX3330 induced attenuation of MCP-1 and RAGE/NF-κB pathway decreases M1 macrophage and promotes M2 macrophage polarization, which then in turn decreases neuroinflammatory factor production. However, the interconnections between M2 polarization and APX3330 induced regulation of neuroinflammation and WM remodeling leading to beneficial effects after stroke in T1DM rats are not completely understood and further studies are warranted.

We acknowledge that APX3330 may have conflicting results in DM versus non-DM stroke. For instance, using an APE1 conditional knockout mouse line, it has been recently demonstrated that the deletion of APE1 significantly increases ischemic lesion volume, worsens neurological functional recovery, impairs neuronal communication and aggravates demyelination as well as neuronal and oligodendrocyte degeneration in non-DM mice subjected to stroke [77]. However, the present study shows that APE1/Ref-1 redox inhibition via APX3330 treatment in DM stroke rats exerts neuroprotective and neurorestorative effects. These differences could be attributed at least in part to the role of VEGF signaling which differs between DM and non-DM stroke [30]. A robust increase in VEGF post ischemic injury to the brain correlated with aggravated BBB disruption in DM stroke mice [30]. Attenuation of VEGF after stroke significantly decreased BBB permeability, prevented the loss of synaptic structure in the IBZ, and promoted neurological functional recovery after stroke in DM mice, however, in non-DM stroke mice; attenuation of VEGF had adverse effects [30]. It has been previously demonstrated that in the acute phase of stroke in non-DM rodents, administration of VEGF enhances cerebral microvascular perfusion, increases BBB leakage, induces cerebral hemorrhage and expands infarction volume after stroke [78]. Our previous study has reported that at 1 day after stroke, VEGF is significantly increased in the ischemic brain of DM rats compared to non-DM rats [79]. Therefore, particularly in DM stroke, VEGF signaling can potentially induce extensive vascular damage [80, 81]. In this study, our data indicate that APX3330 decreases VEGF expression in the IBZ compared to control T1DM stroke rats. Since APE1/Ref-1 redox inhibition can affect multiple signaling pathways including VEGF, effects of APX3330 are likely to be different in DM stroke compared to non-DM stroke and further studies are warranted.

While the molecular mechanisms of APX3330 induced neuroprotective and neurorestorative effects in T1DM stroke rats are still unclear, this proof-of-concept study shows that APX3330 can significantly improve neurological functional outcome, decrease lesion volume, decrease BBB permeability, regulate inflammatory responses and promote white matter remodeling after stroke in T1DM rats.

### Conclusions

APX3330 treatment of stroke significantly improves neurological functional outcome and decreases ischemic lesion volume and BBB permeability in T1DM rats. APX3330 treatment significantly decreases VEGF, PAI-1, RAGE, MCP1 and MMP9 levels in ischemic brain which may contribute to decreasing dysfunctional angiogenesis, inflammation in T1DM stroke rats. APX3330 significantly increases myelin density, oligo-dendrogenesis and synaptic plasticity after stroke in T1DM rats. Therefore, APX3330 treatment derived neuroprotective and neurorestorative effects in T1DM stroke rats may be attributed at least in part to attenuation of dysfunctional angiogenesis, decrease in pro-inflammatory factors and increased white matter remodeling.

## References

[1] Mast H, Thompson JL, Lee SH, Mohr JP, Sacco RL (1995). Hypertension and diabetes mellitus as determinants of multiple lacunar infarcts. Stroke, 26: 30-33 7839393 10.1161/01.str.26.1.30

[2] Li W, Prakash R, Kelly-Cobbs AI, Ogbi S, Kozak A, El-Remessy AB, et al (2010). Adaptive cerebral neovascularization in a model of type 2 diabetes: relevance to focal cerebral ischemia. Diabetes, 59: 228-235 19808897 10.2337/db09-0902 PMC2797926

[3] Capes SE, Hunt D, Malmberg K, Pathak P, Gerstein HC (2001). Stress hyperglycemia and prognosis of stroke in nondiabetic and diabetic patients: a systematic overview. Stroke, 32: 2426-2432 11588337 10.1161/hs1001.096194

[4] Nakaji K, Ihara M, Takahashi C, Itohara S, Noda M, Takahashi R, et al (2006). Matrix metalloproteinase-2 plays a critical role in the pathogenesis of white matter lesions after chronic cerebral hypoperfusion in rodents. Stroke, 37: 2816-2823 17008622 10.1161/01.STR.0000244808.17972.55

[5] Chen J, Cui X, Zacharek A, Cui Y, Roberts C, Chopp M (2011). White matter damage and the effect of matrix metalloproteinases in type 2 diabetic mice after stroke. Stroke, 42: 445-452 21193743 10.1161/STROKEAHA.110.596486 PMC3108495

[6] Singh B, Singh V, Krishnan A, Koshy K, Martinez JA, Cheng C, et al (2014). Regeneration of diabetic axons is enhanced by selective knockdown of the PTEN gene. Brain, 137: 1051-1067 24578546 10.1093/brain/awu031 PMC3959560

[7] Chen J, Venkat P, Zacharek A, Chopp M (2014). Neurorestorative Therapy for Stroke. Front Hum Neurosci, 8 25018718 10.3389/fnhum.2014.00382 PMC4072966

[8] Rosenberg GA, Navratil M, Barone F, Feuerstein G (1996). Proteolytic cascade enzymes increase in focal cerebral ischemia in rat. J Cereb Blood Flow Metab, 16: 360-366 8621740 10.1097/00004647-199605000-00002

[9] Wallin A, Sjogren M, Edman A, Blennow K, Regland B (2000). Symptoms, vascular risk factors and blood-brain barrier function in relation to CT white-matter changes in dementia. Eur Neurol, 44: 229-235 11096223 10.1159/000008242

[10] Asahi M, Wang X, Mori T, Sumii T, Jung JC, Moskowitz MA, et al (2001). Effects of matrix metalloproteinase-9 gene knock-out on the proteolysis of blood-brain barrier and white matter components after cerebral ischemia. J Neurosci, 21: 7724-7732 11567062 10.1523/JNEUROSCI.21-19-07724.2001 PMC6762894

[11] Barile GR, Schmidt AM (2007). RAGE and its ligands in retinal disease. Curr Mol Med, 7: 758-765 18331234 10.2174/156652407783220778

[12] Maillard-Lefebvre H, Boulanger E, Daroux M, Gaxatte C, Hudson BI, Lambert M (2009). Soluble receptor for advanced glycation end products: a new biomarker in diagnosis and prognosis of chronic inflammatory diseases. Rheumatology (Oxford), 48: 1190-1196 19589888 10.1093/rheumatology/kep199

[13] Ye X, Chopp M, Liu X, Zacharek A, Cui X, Yan T, et al (2011). Niaspan reduces high-mobility group box 1/receptor for advanced glycation endproducts after stroke in type-1 diabetic rats. Neuroscience, 190: 339-345 21683770 10.1016/j.neuroscience.2011.06.004 PMC3260534

[14] Emsley HC, Smith CJ, Gavin CM, Georgiou RF, Vail A, Barberan EM, et al (2003). An early and sustained peripheral inflammatory response in acute ischaemic stroke: relationships with infection and atherosclerosis. J Neuroimmunol, 139: 93-101 12799026 10.1016/s0165-5728(03)00134-6

[15] Gelderblom M, Leypoldt F, Steinbach K, Behrens D, Choe CU, Siler DA, et al (2009). Temporal and spatial dynamics of cerebral immune cell accumulation in stroke. Stroke, 40: 1849-1857 19265055 10.1161/STROKEAHA.108.534503

[16] Iadecola C, Anrather J (2011). The immunology of stroke: from mechanisms to translation. Nat Med, 17: 796-808 21738161 10.1038/nm.2399 PMC3137275

[17] Hu X, Li P, Guo Y, Wang H, Leak RK, Chen S, et al (2012). Microglia/macrophage polarization dynamics reveal novel mechanism of injury expansion after focal cerebral ischemia. Stroke, 43: 3063-3070 22933588 10.1161/STROKEAHA.112.659656

[18] Yilmaz G, Granger DN (2010). Leukocyte recruitment and ischemic brain injury. Neuromolecular Med, 12: 193-204 19579016 10.1007/s12017-009-8074-1 PMC2878882

[19] Kelley MR, Georgiadis MM, Fishel ML (2012). APE1/Ref-1 Role in Redox Signaling: Translational Applications of Targeting the Redox Function of the DNA Repair/Redox Protein APE1/Ref-1. Curr Mol Pharmacol, 5: 36-53 22122463 10.2174/1874467211205010036 PMC3319314

[20] Thakur S, Sarkar B, Cholia RP, Gautam N, Dhiman M, Mantha AK (2014). APE1/Ref-1 as an emerging therapeutic target for various human diseases: phytochemical modulation of its functions. Exp Mol Med, 46: e106 25033834 10.1038/emm.2014.42 PMC4119211

[21] J Cereb Blood Flow MetabKelley MR, Fehrenbacher JC (2017). Challenges and opportunities identifying therapeutic targets for chemotherapy-induced peripheral neuropathy resulting from oxidative DNA damage. Neural Regen Res, 12: 72-74 28250749 10.4103/1673-5374.198986 PMC5319244

[22] Shah F, Logsdon D, Messmann RA, Fehrenbacher JC, Fishel ML, Kelley MR (2017). Exploiting the Ref-1-APE1 node in cancer signaling and other diseases: from bench to clinic. NPJ Precis Oncol, 1: 19 28825044 10.1038/s41698-017-0023-0 PMC5558897

[23] Nurmi A, Lindsberg PJ, Koistinaho M, Zhang W, Juettler E, Karjalainen-Lindsberg ML, et al (2004). Nuclear factor-kappaB contributes to infarction after permanent focal ischemia. Stroke, 35: 987-991 14988572 10.1161/01.STR.0000120732.45951.26

[24] Ceulemans AG, Zgavc T, Kooijman R, Hachimi-Idrissi S, Sarre S, Michotte Y (2010). The dual role of the neuroinflammatory response after ischemic stroke: modulatory effects of hypothermia. J Neuroinflammation, 7: 74 21040547 10.1186/1742-2094-7-74 PMC2988764

[25] Ning R, Chopp M, Zacharek A, Yan T, Zhang C, Roberts C, et al (2014). Neamine induces neuroprotection after acute ischemic stroke in type one diabetic rats. Neuroscience, 257: 76-85 24211797 10.1016/j.neuroscience.2013.10.071 PMC3889124

[26] Geng J, Wang L, Qu M, Song Y, Lin X, Chen Y, et al (2017). Endothelial progenitor cells transplantation attenuated blood-brain barrier damage after ischemia in diabetic mice via HIF-1α. Stem Cell Res Ther, 8: 163 28697748 10.1186/s13287-017-0605-3 PMC5505148

[27] Xiao H, Gu Z, Wang G, Zhao T (2013). The Possible Mechanisms Underlying the Impairment of HIF-1α Pathway Signaling in Hyperglycemia and the Beneficial Effects of Certain Therapies. Int J Med Sci, 10: 1412-1421 23983604 10.7150/ijms.5630 PMC3752727

[28] Yan J, Zhang Z, Shi H (2012). HIF-1 is involved in high glucose-induced paracellular permeability of brain endothelial cells. Cell Mol Life Sci, 69: 115-128 21617913 10.1007/s00018-011-0731-5 PMC11115066

[29] Ergul A, Abdelsaid M, Fouda AY, Fagan SC (2014). Cerebral neovascularization in diabetes: implications for stroke recovery and beyond. J Cereb Blood Flow Metab, 34: 553-563 24496174 10.1038/jcbfm.2014.18 PMC3982092

[30] Reeson P, Tennant KA, Gerrow K, Wang J, Weiser Novak S, Thompson K, et al (2015). Delayed inhibition of VEGF signaling after stroke attenuates blood-brain barrier breakdown and improves functional recovery in a comorbidity-dependent manner. J Neurosci, 35: 5128-5143 25834040 10.1523/JNEUROSCI.2810-14.2015 PMC6705411

[31] Jiang A, Gao H, Kelley MR, Qiao X (2011). Inhibition of APE1/Ref-1 redox activity with APX3330 blocks retinal angiogenesis in vitro and in vivo. Vision Res, 51: 93-100 20937296 10.1016/j.visres.2010.10.008 PMC3010438

[32] Like AA, Rossini AA (1976). Streptozotocin-induced pancreatic insulitis: new model of diabetes mellitus. Science, 193: 415-417 180605 10.1126/science.180605

[33] Chen J, Li Y, Wang L, Zhang Z, Lu D, Lu M, et al (2001). Therapeutic benefit of intravenous administration of bone marrow stromal cells after cerebral ischemia in rats. Stroke, 32: 1005-1011 11283404 10.1161/01.str.32.4.1005

[34] Chen H, Chopp M, Zhang ZG, Garcia JH (1992). The effect of hypothermia on transient middle cerebral artery occlusion in the rat. J Cereb Blood Flow Metab, 12: 621-628 1618941 10.1038/jcbfm.1992.86

[35] Rogers DC, Campbell CA, Stretton JL, Mackay KB (1997). Correlation Between Motor Impairment and Infarct Volume After Permanent and Transient Middle Cerebral Artery Occlusion in the Rat. Stroke, 28: 2060-2066 9341719 10.1161/01.str.28.10.2060

[36] Schaar KL, Brenneman MM, Savitz SI (2010). Functional assessments in the rodent stroke model. Exp Transl Stroke Med, 2: 13 20642841 10.1186/2040-7378-2-13 PMC2915950

[37] Swanson RA, Morton MT, Tsao-Wu G, Savalos RA, Davidson C, Sharp FR (1990). A semiautomated method for measuring brain infarct volume. J Cereb Blood Flow Metab, 10: 290-293 1689322 10.1038/jcbfm.1990.47

[38] Yan T, Venkat P, Ye X, Chopp M, Zacharek A, Ning R, et al (2014). HUCBCs Increase Angiopoietin 1 and Induce Neurorestorative Effects after Stroke in T1DM Rats. CNS Neurosci Ther, 20: 935-944 25042092 10.1111/cns.12307 PMC4180763

[39] Yan T, Venkat P, Chopp M, Zacharek A, Ning R, Cui Y, et al (2015). Neurorestorative Therapy of Stroke in Type 2 Diabetes Mellitus Rats Treated With Human Umbilical Cord Blood Cells. Stroke, 46: 2599-2606 26243222 10.1161/STROKEAHA.115.009870 PMC4550506

[40] Chen J, Ning R, Zacharek A, Cui C, Cui X, Yan T, et al (2016). MiR-126 Contributes to Human Umbilical Cord Blood Cell-Induced Neurorestorative Effects After Stroke in Type-2 Diabetic Mice. Stem cells, 34: 102-113 26299579 10.1002/stem.2193 PMC4713352

[41] Chen J, Zhang ZG, Li Y, Wang Y, Wang L, Jiang H, et al (2003). Statins induce angiogenesis, neurogenesis, and synaptogenesis after stroke. Ann Neurol, 53: 743-751 12783420 10.1002/ana.10555

[42] Ueno Y, Chopp M, Zhang L, Buller B, Liu Z, Lehman NL, et al (2012). Axonal outgrowth and dendritic plasticity in the cortical peri-infarct area after experimental stroke. Stroke, 43: 2221-2228 22618383 10.1161/STROKEAHA.111.646224 PMC3404219

[43] Taylor AM, Blurton-Jones M, Rhee SW, Cribbs DH, Cotman CW, Jeon NL (2005). A microfluidic culture platform for CNS axonal injury, regeneration and transport. Nat Methods, 2: 599-605 16094385 10.1038/nmeth777 PMC1558906

[44] Ye X, Chopp M, Cui X, Zacharek A, Cui Y, Yan T, et al (2011). Niaspan enhances vascular remodeling after stroke in type 1 diabetic rats. Exp Neurol, 232: 299-308 21963653 10.1016/j.expneurol.2011.09.022 PMC3265018

[45] Hori S, Ohtsuki S, Hosoya K, Nakashima E, Terasaki T (2004). A pericyte-derived angiopoietin-1 multimeric complex induces occludin gene expression in brain capillary endothelial cells through Tie-2 activation in vitro. J Neurochem, 89: 503-513 15056293 10.1111/j.1471-4159.2004.02343.x

[46] Megherbi SE, Milan C, Minier D, Couvreur G, Osseby GV, Tilling K, et al (2003). Association between diabetes and stroke subtype on survival and functional outcome 3 months after stroke: data from the European BIOMED Stroke Project. Stroke, 34: 688-694 12624292 10.1161/01.STR.0000057975.15221.40

[47] Sahin I, Alkan A, Keskin L, Cikim A, Karakas HM, Firat AK, et al (2008). Evaluation of in vivo cerebral metabolism on proton magnetic resonance spectroscopy in patients with impaired glucose tolerance and type 2 diabetes mellitus. J Diabetes Complications, 22: 254-260 18413166 10.1016/j.jdiacomp.2007.03.007

[48] Leys D, Englund E, Del Ser T, Inzitari D, Fazekas F, Bornstein N, et al (1999). White matter changes in stroke patients. Relationship with stroke subtype and outcome. Eur Neurol, 42: 67-75 10473977 10.1159/000069414

[49] Arai K, Lo EH (2009). Oligovascular signaling in white matter stroke. Biol Pharm Bull, 32: 1639-1644 19801821 10.1248/bpb.32.1639 PMC3691964

[50] Mandai K, Matsumoto M, Kitagawa K, Matsushita K, Ohtsuki T, Mabuchi T, et al (1997). Ischemic damage and subsequent proliferation of oligodendrocytes in focal cerebral ischemia. Neuroscience, 77: 849-861 9070757

[51] Chen J, Chopp M (2006). Neurorestorative treatment of stroke: Cell and pharmacological approaches. NeuroRx, 3: 466-473 17012060 10.1016/j.nurx.2006.07.007 PMC2790719

[52] Venkat P, Chopp M, Chen J (2017). Blood-Brain Barrier Disruption, Vascular Impairment, and Ischemia/Reperfusion Damage in Diabetic Stroke. J Am Heart Assoc, 6 10.1161/JAHA.117.005819 PMC5669184 28572280

[53] Kimura-Ohba S, Yang Y (2016). Oxidative DNA Damage Mediated by Intranuclear MMP Activity Is Associated with Neuronal Apoptosis in Ischemic Stroke. Oxid Med Cell Longev, 2016: 9 10.1155/2016/6927328 PMC4748094 26925194

[54] Gao D, Huang T, Jiang X, Hu S, Zhang L, Fei Z (2014). Resveratrol protects primary cortical neuron cultures from transient oxygen-glucose deprivation by inhibiting MMP-9. Mol Med Rep, 9: 2197-2204 24682241 10.3892/mmr.2014.2086

[55] Trost S, Pratley R, Sobel B (2006). Impaired fibrinolysis and risk for cardiovascular disease in the metabolic syndrome and type 2 diabetes. Curr Diab Rep, 6: 47-54 16522281 10.1007/s11892-006-0052-5

[56] Grant PJ (2007). Diabetes mellitus as a prothrombotic condition. J Intern Med, 262: 157-172 17645584 10.1111/j.1365-2796.2007.01824.x

[57] Jankun J, Al-Senaidy A, Skrzypczak-Jankun E (2012). Can inactivators of plasminogen activator inhibitor alleviate the burden of obesity and diabetes? (Review). Int J Mol Med, 29: 3-11 21993838 10.3892/ijmm.2011.810

[58] Kaji H (2016). Adipose Tissue-Derived Plasminogen Activator Inhibitor-1 Function and Regulation. Compr Physiol, 6: 1873-1896 27783862 10.1002/cphy.c160004

[59] Mertens I, Verrijken A, Michiels JJ, Van der Planken M, Ruige JB, Van Gaal LF (2006). Among inflammation and coagulation markers, PAI-1 is a true component of the metabolic syndrome. Int J Obes, 30: 1308-1314 10.1038/sj.ijo.0803189 16389265

[60] Vinagre I, Sanchez-Quesada JL, Sanchez-Hernandez J, Santos D, Ordonez-Llanos J, De Leiva A, et al (2014). Inflammatory biomarkers in type 2 diabetic patients: effect of glycemic control and impact of LDL subfraction phenotype. Cardiovasc Diabetol, 13: 34 24495560 10.1186/1475-2840-13-34 PMC3922962

[61] Menegazzo L, Poncina N, Albiero M, Menegolo M, Grego F, Avogaro A, et al (2015). Diabetes modifies the relationships among carotid plaque calcification, composition and inflammation. Atherosclerosis, 241: 533-538 26093886 10.1016/j.atherosclerosis.2015.06.013

[62] Nazir N, Siddiqui K, Al-Qasim S, Al-Naqeb D (2014). Meta-analysis of diabetic nephropathy associated genetic variants in inflammation and angiogenesis involved in different biochemical pathways. BMC Med Genet, 15: 103 25280384 10.1186/s12881-014-0103-8 PMC4411872

[63] Bose S, Cho J (2013). Role of chemokine CCL2 and its receptor CCR2 in neurodegenerative diseases. Arch Pharm Res, 36: 1039-1050 23771498 10.1007/s12272-013-0161-z

[64] Morancho A, Ma F, Barcelo V, Giralt D, Montaner J, Rosell A (2015). Impaired vascular remodeling after endothelial progenitor cell transplantation in MMP9-deficient mice suffering cortical cerebral ischemia. J Cereb Blood Flow Metab, 35: 1547-1551 26219597 10.1038/jcbfm.2015.180 PMC4640313

[65] Chaturvedi M, Kaczmarek L (2014). MMP-9 Inhibition: a Therapeutic Strategy in Ischemic Stroke. Mol Neurobiol, 49: 563-573 24026771 10.1007/s12035-013-8538-z PMC3918117

[66] Jalal FY, Yang Y, Thompson J, Lopez AC, Rosenberg GA (2012). Myelin loss associated with neuroinflammation in hypertensive rats. Stroke, 43: 1115-1122 22363061 10.1161/STROKEAHA.111.643080 PMC3598597

[67] Schmidt AM, Yan SD, Yan SF, Stern DM (2001). The multiligand receptor RAGE as a progression factor amplifying immune and inflammatory responses. J Clin Invest, 108: 949-955 11581294 10.1172/JCI14002 PMC200958

[68] Journal of Clinical InvestigationHassid BG, Nair MN, Ducruet AF, Otten ML, Komotar RJ, Pinsky DJ, et al (2009). Neuronal RAGE expression modulates severity of injury following transient focal cerebral ischemia. J Clin Neurosci, 16: 302-306 19071026 10.1016/j.jocn.2007.12.011

[69] Muhammad S, Barakat W, Stoyanov S, Murikinati S, Yang H, Tracey KJ, et al (2008). The HMGB1 receptor RAGE mediates ischemic brain damage. J Neurosci, 28: 12023-12031 19005067 10.1523/JNEUROSCI.2435-08.2008 PMC4597312

[70] Perego C, Fumagalli S, De Simoni M-G (2013). Three-dimensional Confocal Analysis of Microglia/macrophage Markers of Polarization in Experimental Brain Injury. J Vis Exp: 50605 10.3791/50605 PMC3857388 24056862

[71] Jin Q, Cheng J, Liu Y, Wu J, Wang X, Wei S, et al (2014). Improvement of functional recovery by chronic metformin treatment is associated with enhanced alternative activation of microglia/macrophages and increased angiogenesis and neurogenesis following experimental stroke. Brain Behav Immun, 40: 131-142 24632338 10.1016/j.bbi.2014.03.003

[72] Kigerl KA, Gensel JC, Ankeny DP, Alexander JK, Donnelly DJ, Popovich PG (2009). Identification of two distinct macrophage subsets with divergent effects causing either neurotoxicity or regeneration in the injured mouse spinal cord. J Neurosci, 29: 13435-13444 19864556 10.1523/JNEUROSCI.3257-09.2009 PMC2788152

[73] Ylä-Herttuala S, Lipton BA, Rosenfeld ME, Särkioja T, Yoshimura T, Leonard EJ, et al (1991). Expression of monocyte chemoattractant protein 1 in macrophage-rich areas of human and rabbit atherosclerotic lesions. Proc Natl Acad Sci U S A, 88: 5252-5256 2052604 10.1073/pnas.88.12.5252 PMC51850

[74] Hughes PM, Allegrini PR, Rudin M, Perry VH, Mir AK, Wiessner C (2002). Monocyte chemoattractant protein-1 deficiency is protective in a murine stroke model. J Cereb Blood Flow Metab, 22: 308-317 11891436 10.1097/00004647-200203000-00008

[75] Jin X, Yao T, Zhou Z, Zhu J, Zhang S, et al (2015). Advanced Glycation End Products Enhance Macrophages Polarization into M1 Phenotype through Activating RAGE/NF-κB Pathway. Biomed Res Int, 2015: 12 10.1155/2015/732450 PMC4465680 26114112

[76] Dong X, Song Y-N, Liu W-G, Guo X-L (2009). MMP-9, a Potential Target for Cerebral Ischemic Treatment. Curr Neuropharmacol, 7: 269-275 20514206 10.2174/157015909790031157 PMC2811860

[77] Stetler RA, Gao Y, Leak RK, Weng Z, Shi Y, Zhang L, et al (2016). APE1/Ref-1 facilitates recovery of gray and white matter and neurological function after mild stroke injury. Proc Natl Acad Sci U S A, 113: E3558-3567 27274063 10.1073/pnas.1606226113 PMC4922172

[78] Zhang ZG, Zhang L, Jiang Q, Zhang R, Davies K, Powers C, et al (2000). VEGF enhances angiogenesis and promotes blood-brain barrier leakage in the ischemic brain. J Clin Invest, 106: 829-838 11018070 10.1172/JCI9369 PMC517814

[79] Yan T, Venkat P, Chopp M, Zacharek A, Ning R, Roberts C, et al (2016). Neurorestorative Responses to Delayed Human Mesenchymal Stromal Cells Treatment of Stroke in Type 2 Diabetic Rats. Stroke, 47: 2850-2858 27729575 10.1161/STROKEAHA.116.014686 PMC5134897

[80] Kolluru GK, Bir SC, Kevil CG (2012). Endothelial dysfunction and diabetes: effects on angiogenesis, vascular remodeling, and wound healing. Int J Vasc Med, 2012: 918267 22611498 10.1155/2012/918267 PMC3348526

[81] Prakash R, Li W, Qu Z, Johnson MA, Fagan SC, Ergul A (2013). Vascularization pattern after ischemic stroke is different in control versus diabetic rats: relevance to stroke recovery. Stroke, 44: 2875-2882 23920018 10.1161/STROKEAHA.113.001660 PMC3827629

